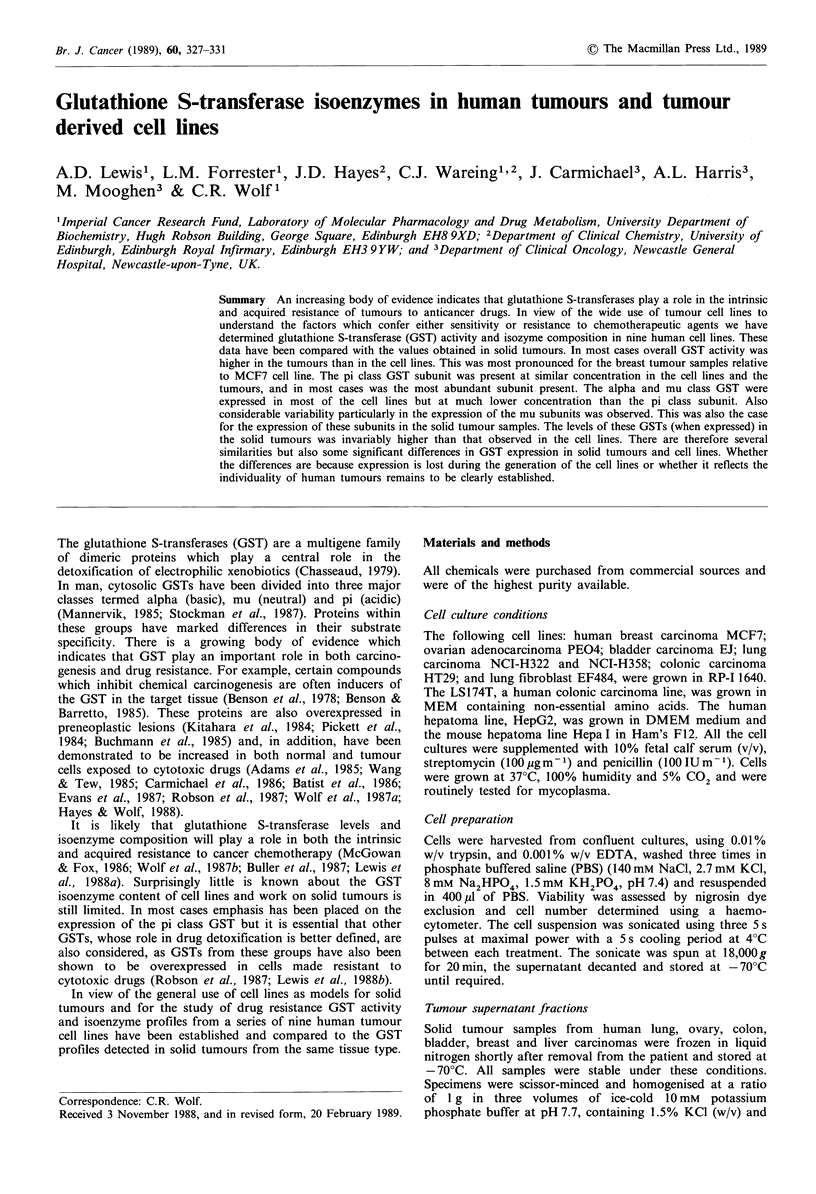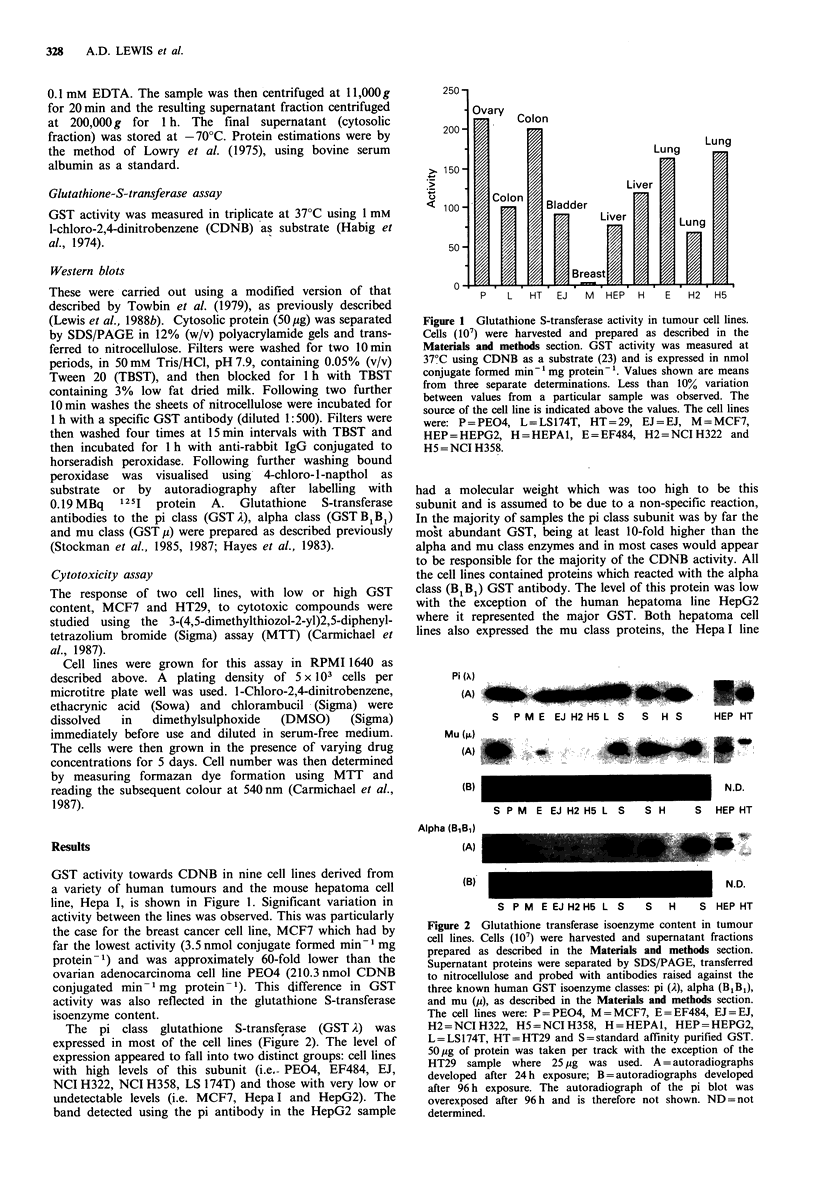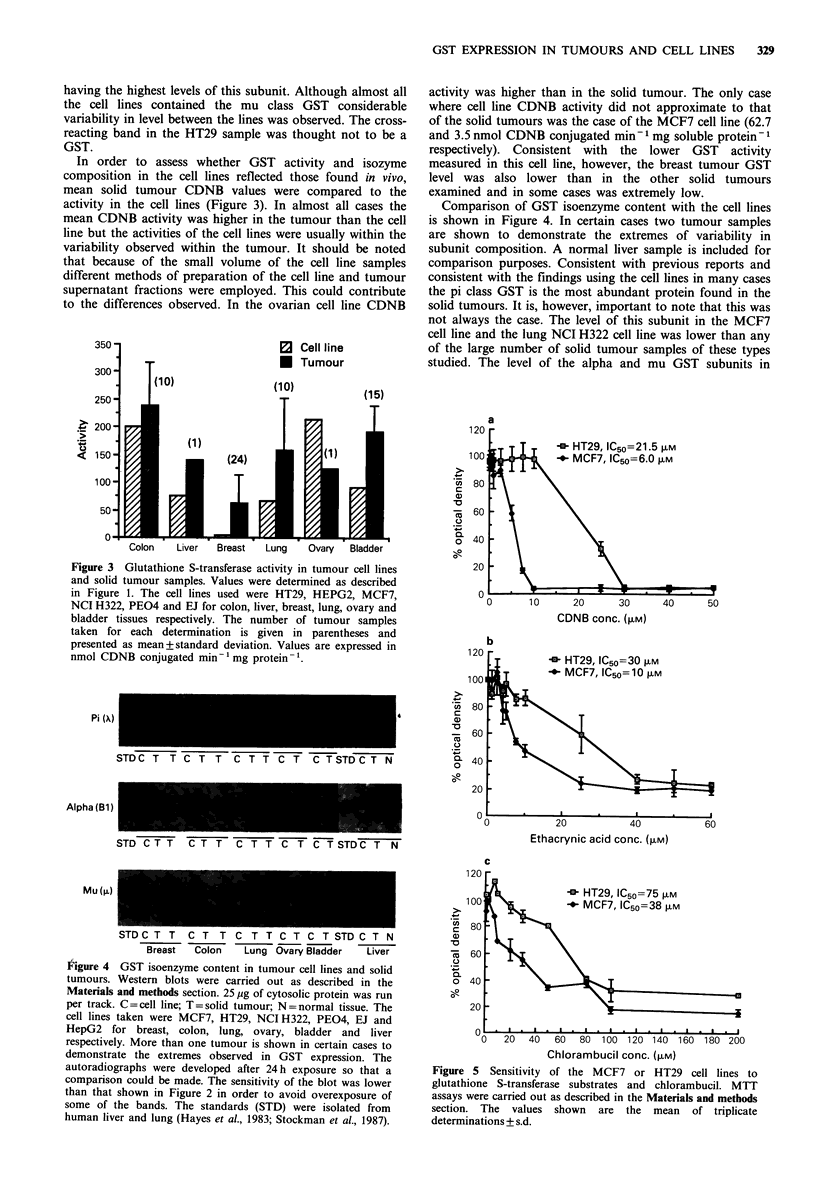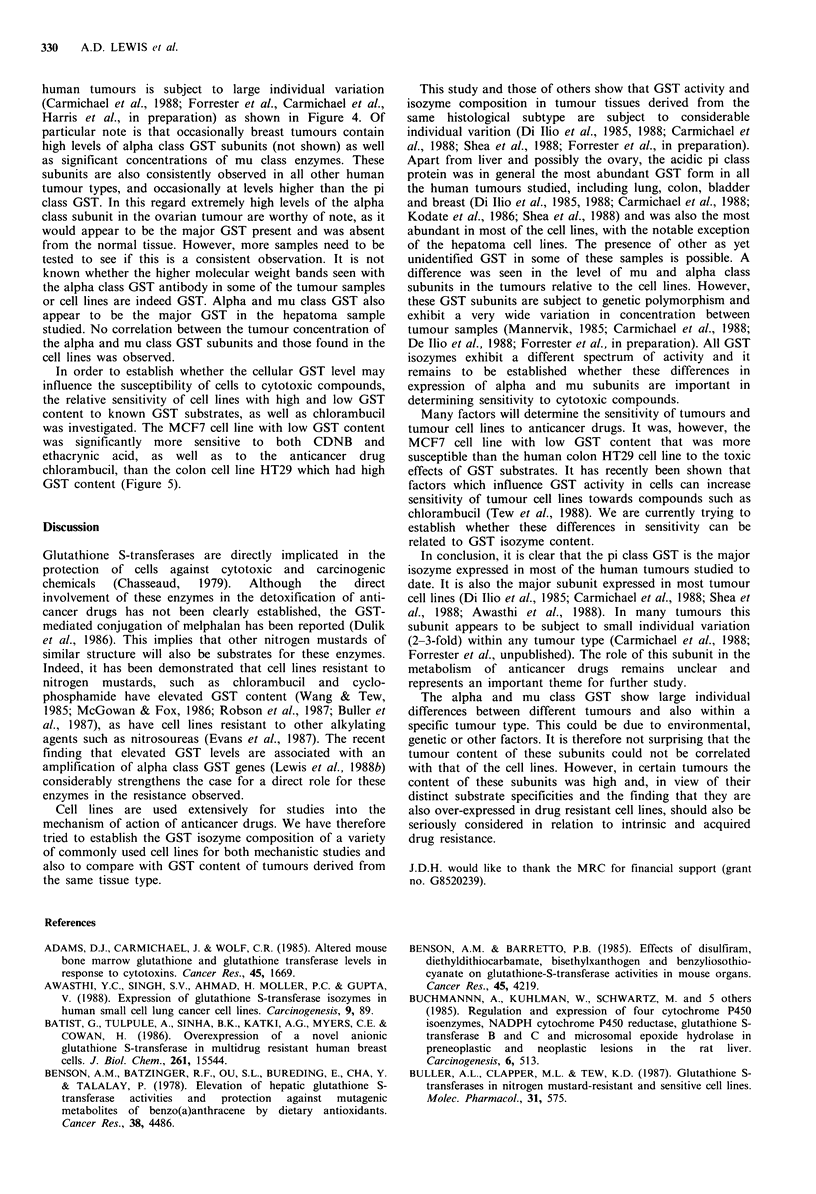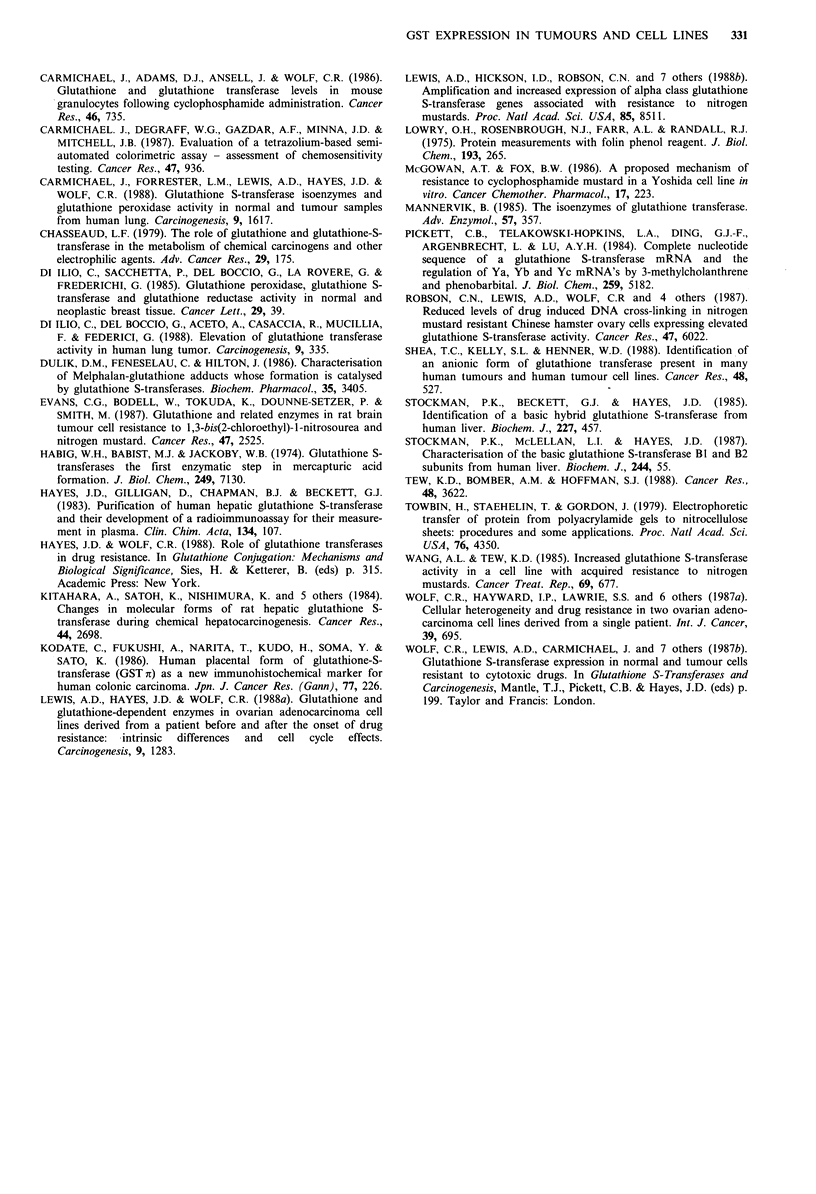# Glutathione S-transferase isoenzymes in human tumours and tumour derived cell lines.

**DOI:** 10.1038/bjc.1989.280

**Published:** 1989-09

**Authors:** A. D. Lewis, L. M. Forrester, J. D. Hayes, C. J. Wareing, J. Carmichael, A. L. Harris, M. Mooghen, C. R. Wolf

**Affiliations:** Imperial Cancer Research Fund, Laboratory of Molecular Pharmacology and Drug Metabolism, University Department of Biochemistry, Edinburgh, UK.

## Abstract

**Images:**


Br. J. Cancer (1989), 60, 327–331 © The Macmillan Press Ltd., 1989

### Glutathione S-transferase isoenzymes in human tumours and tumour derived cell lines

A.D. Lewis[1], L.M. Forrester[1], J.D. Hayes[2], C.J. Wareing[1,2], J. Carmichael[3], A.L. Harris[3], M. Mooghen[3] & C.R. Wolf[1]

[1]*Imperial Cancer Research Fund, Laboratory of Molecular Pharmacology and Drug Metabolism, University Department of Biochemistry, Hugh Robson Building, George Square, Edinburgh EH8 9XD;* [2]*Department of Clinical Chemistry, University of Edinburgh, Edinburgh Royal Infirmary, Edinburgh EH3 9YW; and* [3]*Department of Clinical Oncology, Newcastle General Hospital, Newcastle-upon-Tyne, UK.*

**Summary** An increasing body of evidence indicates that glutathione S-transferases play a role in the intrinsic and acquired resistance of tumours to anticancer drugs. In view of the wide use of tumour cell lines to understand the factors which confer either sensitivity or resistance to chemotherapeutic agents we have determined glutathione S-transferase (GST) activity and isozyme composition in nine human cell lines. These data have been compared with the values obtained in solid tumours. In most cases overall GST activity was higher in the tumours than in the cell lines. This was most pronounced for the breast tumour samples relative to MCF7 cell line. The pi class GST subunit was present at similar concentration in the cell lines and the tumours, and in most cases was the most abundant subunit present. The alpha and mu class GST were expressed in most of the cell lines but at much lower concentration than the pi class subunit. Also considerable variability particularly in the expression of the mu subunits was observed. This was also the case for the expression of these subunits in the solid tumour samples. The levels of these GSTs (when expressed) in the solid tumours was invariably higher than that observed in the cell lines. There are therefore several similarities but also some significant differences in GST expression in solid tumours and cell lines. Whether the differences are because expression is lost during the generation of the cell lines or whether it reflects the individuality of human tumours remains to be clearly established.

The glutathione S-transferases (GST) are a multigene family of dimeric proteins which play a central role in the detoxification of electrophilic xenobiotics (Chasseaud, 1979). In man, cytosolic GSTs have been divided into three major classes termed alpha (basic), mu (neutral) and pi (acidic) (Mannervik, 1985; Stockman *et al.*, 1987). Proteins within these groups have marked differences in their substrate specificity. There is a growing body of evidence which indicates that GST play an important role in both carcinogenesis and drug resistance. For example, certain compounds which inhibit chemical carcinogenesis are often inducers of the GST in the target tissue (Benson *et al.*, 1978; Benson & Barretto, 1985). These proteins are also overexpressed in preneoplastic lesions (Kitahara *et al.*, 1984; Pickett *et al.*, 1984; Buchmann *et al.*, 1985) and, in addition, have been demonstrated to be increased in both normal and tumour cells exposed to cytotoxic drugs (Adams *et al.*, 1985; Wang & Tew, 1985; Carmichael *et al.*, 1986; Batist *et al.*, 1986; Evans *et al.*, 1987; Robson *et al.*, 1987; Wolf *et al.*, 1987*a*; Hayes & Wolf, 1988).

It is likely that glutathione S-transferase levels and isoenzyme composition will play a role in both the intrinsic and acquired resistance to cancer chemotherapy (McGowan & Fox, 1986; Wolf *et al.*, 1987*b*; Buller *et al.*, 1987; Lewis *et al.*, 1988*a*). Surprisingly little is known about the GST isoenzyme content of cell lines and work on solid tumours is still limited. In most cases emphasis has been placed on the expression of the pi class GST but it is essential that other GSTs, whose role in drug detoxification is better defined, are also considered, as GSTs from these groups have also been shown to be overexpressed in cells made resistant to cytotoxic drugs (Robson *et al.*, 1987; Lewis *et al.*, 1988*b*).

In view of the general use of cell lines as models for solid tumours and for the study of drug resistance GST activity and isoenzyme profiles from a series of nine human tumour cell lines have been established and compared to the GST profiles detected in solid tumours from the same tissue type.

Correspondence: C.R. Wolf.
Received 3 November 1988, and in revised form, 20 February 1989.

### Materials and methods

All chemicals were purchased from commercial sources and were of the highest purity available.

### *Cell culture conditions*

The following cell lines: human breast carcinoma MCF7; ovarian adenocarcinoma PEO4; bladder carcinoma EJ; lung carcinoma NCI-H322 and NCI-H358; colonic carcinoma HT29; and lung fibroblast EF484, were grown in RP-I 1640. The LS174T, a human colonic carcinoma line, was grown in MEM containing non-essential amino acids. The human hepatoma line, HepG2, was grown in DMEM medium and the mouse hepatoma line Hepa I in Ham's F12. All the cell cultures were supplemented with 10% fetal calf serum (v/v), streptomycin (100 $\mu g\,m^{-1}$) and penicillin (100 $IU\,m^{-1}$). Cells were grown at 37°C, 100% humidity and 5% $CO_2$ and were routinely tested for mycoplasma.

### *Cell preparation*

Cells were harvested from confluent cultures, using 0.01% w/v trypsin, and 0.001% w/v EDTA, washed three times in phosphate buffered saline (PBS) (140 mM NaCl, 2.7 mM KCl, 8 mM $Na_2HPO_4$, 1.5 mM $KH_2PO_4$, pH 7.4) and resuspended in 400 $\mu l$ of PBS. Viability was assessed by nigrosin dye exclusion and cell number determined using a haemocytometer. The cell suspension was sonicated using three 5 s pulses at maximal power with a 5 s cooling period at 4°C between each treatment. The sonicate was spun at 18,000 *g* for 20 min, the supernatant decanted and stored at −70°C until required.

### *Tumour supernatant fractions*

Solid tumour samples from human lung, ovary, colon, bladder, breast and liver carcinomas were frozen in liquid nitrogen shortly after removal from the patient and stored at −70°C. All samples were stable under these conditions. Specimens were scissor-minced and homogenised at a ratio of 1 g in three volumes of ice-cold 10 mM potassium phosphate buffer at pH 7.7, containing 1.5% KCl (w/v) and

0.1 mM EDTA. The sample was then centrifuged at 11,000 *g* for 20 min and the resulting supernatant fraction centrifuged at 200,000 *g* for 1 h. The final supernatant (cytosolic fraction) was stored at −70°C. Protein estimations were by the method of Lowry *et al.* (1975), using bovine serum albumin as a standard.

### *Glutathione-S-transferase assay*

GST activity was measured in triplicate at 37°C using 1 mM 1-chloro-2,4-dinitrobenzene (CDNB) as substrate (Habig *et al.*, 1974).

### *Western blots*

These were carried out using a modified version of that described by Towbin *et al.* (1979), as previously described (Lewis *et al.*, 1988*b*). Cytosolic protein (50 μg) was separated by SDS/PAGE in 12% (w/v) polyacrylamide gels and transferred to nitrocellulose. Filters were washed for two 10 min periods, in 50 mM Tris/HCl, pH 7.9, containing 0.05% (v/v) Tween 20 (TBST), and then blocked for 1 h with TBST containing 3% low fat dried milk. Following two further 10 min washes the sheets of nitrocellulose were incubated for 1 h with a specific GST antibody (diluted 1:500). Filters were then washed four times at 15 min intervals with TBST and then incubated for 1 h with anti-rabbit IgG conjugated to horseradish peroxidase. Following further washing bound peroxidase was visualised using 4-chloro-1-napthol as substrate or by autoradiography after labelling with 0.19 MBq $^{125}I$ protein A. Glutathione S-transferase antibodies to the pi class (GST λ), alpha class (GST $B_1B_1$) and mu class (GST μ) were prepared as described previously (Stockman *et al.*, 1985, 1987; Hayes *et al.*, 1983).

### *Cytotoxicity assay*

The response of two cell lines, with low or high GST content, MCF7 and HT29, to cytotoxic compounds were studied using the 3-(4,5-dimethylthiozol-2-yl)2,5-diphenyl-tetrazolium bromide (Sigma) assay (MTT) (Carmichael *et al.*, 1987).

Cell lines were grown for this assay in RPMI 1640 as described above. A plating density of $5 \times 10^3$ cells per microtitre plate well was used. 1-Chloro-2,4-dinitrobenzene, ethacrynic acid (Sowa) and chlorambucil (Sigma) were dissolved in dimethylsulphoxide (DMSO) (Sigma) immediately before use and diluted in serum-free medium. The cells were then grown in the presence of varying drug concentrations for 5 days. Cell number was then determined by measuring formazan dye formation using MTT and reading the subsequent colour at 540 nm (Carmichael *et al.*, 1987).

### Results

GST activity towards CDNB in nine cell lines derived from a variety of human tumours and the mouse hepatoma cell line, Hepa I, is shown in Figure 1. Significant variation in activity between the lines was observed. This was particularly the case for the breast cancer cell line, MCF7 which had by far the lowest activity (3.5 nmol conjugate formed $min^{-1}$ mg $protein^{-1}$) and was approximately 60-fold lower than the ovarian adenocarcinoma cell line PEO4 (210.3 nmol CDNB conjugated $min^{-1}$ mg $protein^{-1}$). This difference in GST activity was also reflected in the glutathione S-transferase isoenzyme content.

The pi class glutathione S-transferase (GST λ) was expressed in most of the cell lines (Figure 2). The level of expression appeared to fall into two distinct groups: cell lines with high levels of this subunit (i.e. PEO4, EF484, EJ, NCI H322, NCI H358, LS 174T) and those with very low or undetectable levels (i.e. MCF7, Hepa I and HepG2). The band detected using the pi antibody in the HepG2 sample

**Figure 1** Glutathione S-transferase activity in tumour cell lines. Cells ($10^7$) were harvested and prepared as described in the **Materials and methods** section. GST activity was measured at 37°C using CDNB as a substrate (23) and is expressed in nmol conjugate formed $min^{-1}$ mg $protein^{-1}$. Values shown are means from three separate determinations. Less than 10% variation between values from a particular sample was observed. The source of the cell line is indicated above the values. The cell lines were: P = PEO4, L = LS174T, HT = 29, EJ = EJ, M = MCF7, HEP = HEPG2, H = HEPA1, E = EF484, H2 = NCI H322 and H5 = NCI H358.

had a molecular weight which was too high to be this subunit and is assumed to be due to a non-specific reaction, In the majority of samples the pi class subunit was by far the most abundant GST, being at least 10-fold higher than the alpha and mu class enzymes and in most cases would appear to be responsible for the majority of the CDNB activity. All the cell lines contained proteins which reacted with the alpha class ($B_1B_1$) GST antibody. The level of this protein was low with the exception of the human hepatoma line HepG2 where it represented the major GST. Both hepatoma cell lines also expressed the mu class proteins, the Hepa I line

**Figure 2** Glutathione transferase isoenzyme content in tumour cell lines. Cells ($10^7$) were harvested and supernatant fractions prepared as described in the **Materials and methods** section. Supernatant proteins were separated by SDS/PAGE, transferred to nitrocellulose and probed with antibodies raised against the three known human GST isoenzyme classes: pi (λ), alpha ($B_1B_1$), and mu (μ), as described in the **Materials and methods** section. The cell lines were: P = PEO4, M = MCF7, E = EF484, EJ = EJ, H2 = NCI H322, H5 = NCI H358, H = HEPA1, HEP = HEPG2, L = LS174T, HT = HT29 and S = standard affinity purified GST. 50 μg of protein was taken per track with the exception of the HT29 sample where 25 μg was used. A = autoradiographs developed after 24 h exposure; B = autoradiographs developed after 96 h exposure. The autoradiograph of the pi blot was overexposed after 96 h and is therefore not shown. ND = not determined.

having the highest levels of this subunit. Although almost all the cell lines contained the mu class GST considerable variability in level between the lines was observed. The cross-reacting band in the HT29 sample was thought not to be a GST.

In order to assess whether GST activity and isozyme composition in the cell lines reflected those found *in vivo*, mean solid tumour CDNB values were compared to the activity in the cell lines (Figure 3). In almost all cases the mean CDNB activity was higher in the tumour than the cell line but the activities of the cell lines were usually within the variability observed within the tumour. It should be noted that because of the small volume of the cell line samples different methods of preparation of the cell line and tumour supernatant fractions were employed. This could contribute to the differences observed. In the ovarian cell line CDNB activity was higher than in the solid tumour. The only case where cell line CDNB activity did not approximate to that of the solid tumours was the case of the MCF7 cell line (62.7 and 3.5 nmol CDNB conjugated $min^{-1}$ mg soluble $protein^{-1}$ respectively). Consistent with the lower GST activity measured in this cell line, however, the breast tumour GST level was also lower than in the other solid tumours examined and in some cases was extremely low.

Comparison of GST isoenzyme content with the cell lines is shown in Figure 4. In certain cases two tumour samples are shown to demonstrate the extremes of variability in subunit composition. A normal liver sample is included for comparison purposes. Consistent with previous reports and consistent with the findings using the cell lines in many cases the pi class GST is the most abundant protein found in the solid tumours. It is, however, important to note that this was not always the case. The level of this subunit in the MCF7 cell line and the lung NCI H322 cell line was lower than any of the large number of solid tumour samples of these types studied. The level of the alpha and mu GST subunits in

**Figure 3** Glutathione S-transferase activity in tumour cell lines and solid tumour samples. Values were determined as described in Figure 1. The cell lines used were HT29, HEPG2, MCF7, NCI H322, PEO4 and EJ for colon, liver, breast, lung, ovary and bladder tissues respectively. The number of tumour samples taken for each determination is given in parentheses and presented as mean ± standard deviation. Values are expressed in nmol CDNB conjugated $min^{-1}$ mg $protein^{-1}$.

**Figure 4** GST isoenzyme content in tumour cell lines and solid tumours. Western blots were carried out as described in the **Materials and methods** section. 25 μg of cytosolic protein was run per track. C = cell line; T = solid tumour; N = normal tissue. The cell lines taken were MCF7, HT29, NCI H322, PEO4, EJ and HepG2 for breast, colon, lung, ovary, bladder and liver respectively. More than one tumour is shown in certain cases to demonstrate the extremes observed in GST expression. The autoradiographs were developed after 24 h exposure so that a comparison could be made. The sensitivity of the blot was lower than that shown in Figure 2 in order to avoid overexposure of some of the bands. The standards (STD) were isolated from human liver and lung (Hayes *et al.*, 1983; Stockman *et al.*, 1987).

**Figure 5** Sensitivity of the MCF7 or HT29 cell lines to glutathione S-transferase substrates and chlorambucil. MTT assays were carried out as described in the **Materials and methods** section. The values shown are the mean of triplicate determinations ± s.d.

human tumours is subject to large individual variation (Carmichael *et al.*, 1988; Forrester *et al.*, Carmichael *et al.*, Harris *et al.*, in preparation) as shown in Figure 4. Of particular note is that occasionally breast tumours contain high levels of alpha class GST subunits (not shown) as well as significant concentrations of mu class enzymes. These subunits are also consistently observed in all other human tumour types, and occasionally at levels higher than the pi class GST. In this regard extremely high levels of the alpha class subunit in the ovarian tumour are worthy of note, as it would appear to be the major GST present and was absent from the normal tissue. However, more samples need to be tested to see if this is a consistent observation. It is not known whether the higher molecular weight bands seen with the alpha class GST antibody in some of the tumour samples or cell lines are indeed GST. Alpha and mu class GST also appear to be the major GST in the hepatoma sample studied. No correlation between the tumour concentration of the alpha and mu class GST subunits and those found in the cell lines was observed.

In order to establish whether the cellular GST level may influence the susceptibility of cells to cytotoxic compounds, the relative sensitivity of cell lines with high and low GST content to known GST substrates, as well as chlorambucil was investigated. The MCF7 cell line with low GST content was significantly more sensitive to both CDNB and ethacrynic acid, as well as to the anticancer drug chlorambucil, than the colon cell line HT29 which had high GST content (Figure 5).

### Discussion

Glutathione S-transferases are directly implicated in the protection of cells against cytotoxic and carcinogenic chemicals (Chasseaud, 1979). Although the direct involvement of these enzymes in the detoxification of anticancer drugs has not been clearly established, the GST-mediated conjugation of melphalan has been reported (Dulik *et al.*, 1986). This implies that other nitrogen mustards of similar structure will also be substrates for these enzymes. Indeed, it has been demonstrated that cell lines resistant to nitrogen mustards, such as chlorambucil and cyclophosphamide have elevated GST content (Wang & Tew, 1985; McGowan & Fox, 1986; Robson *et al.*, 1987; Buller *et al.*, 1987), as have cell lines resistant to other alkylating agents such as nitrosoureas (Evans *et al.*, 1987). The recent finding that elevated GST levels are associated with an amplification of alpha class GST genes (Lewis *et al.*, 1988*b*) considerably strengthens the case for a direct role for these enzymes in the resistance observed.

Cell lines are used extensively for studies into the mechanism of action of anticancer drugs. We have therefore tried to establish the GST isozyme composition of a variety of commonly used cell lines for both mechanistic studies and also to compare with GST content of tumours derived from the same tissue type.

This study and those of others show that GST activity and isozyme composition in tumour tissues derived from the same histological subtype are subject to considerable individual varition (Di Ilio *et al.*, 1985, 1988; Carmichael *et al.*, 1988; Shea *et al.*, 1988; Forrester *et al.*, in preparation). Apart from liver and possibly the ovary, the acidic pi class protein was in general the most abundant GST form in all the human tumours studied, including lung, colon, bladder and breast (Di Ilio *et al.*, 1985, 1988; Carmichael *et al.*, 1988; Kodate *et al.*, 1986; Shea *et al.*, 1988) and was also the most abundant in most of the cell lines, with the notable exception of the hepatoma cell lines. The presence of other as yet unidentified GST in some of these samples is possible. A difference was seen in the level of mu and alpha class subunits in the tumours relative to the cell lines. However, these GST subunits are subject to genetic polymorphism and exhibit a very wide variation in concentration between tumour samples (Mannervik, 1985; Carmichael *et al.*, 1988; De Ilio *et al.*, 1988; Forrester *et al.*, in preparation). All GST isozymes exhibit a different spectrum of activity and it remains to be established whether these differences in expression of alpha and mu subunits are important in determining sensitivity to cytotoxic compounds.

Many factors will determine the sensitivity of tumours and tumour cell lines to anticancer drugs. It was, however, the MCF7 cell line with low GST content that was more susceptible than the human colon HT29 cell line to the toxic effects of GST substrates. It has recently been shown that factors which influence GST activity in cells can increase sensitivity of tumour cell lines towards compounds such as chlorambucil (Tew *et al.*, 1988). We are currently trying to establish whether these differences in sensitivity can be related to GST isozyme content.

In conclusion, it is clear that the pi class GST is the major isozyme expressed in most of the human tumours studied to date. It is also the major subunit expressed in most tumour cell lines (Di Ilio *et al.*, 1985; Carmichael *et al.*, 1988; Shea *et al.*, 1988; Awasthi *et al.*, 1988). In many tumours this subunit appears to be subject to small individual variation (2–3-fold) within any tumour type (Carmichael *et al.*, 1988; Forrester *et al.*, unpublished). The role of this subunit in the metabolism of anticancer drugs remains unclear and represents an important theme for further study.

The alpha and mu class GST show large individual differences between different tumours and also within a specific tumour type. This could be due to environmental, genetic or other factors. It is therefore not surprising that the tumour content of these subunits could not be correlated with that of the cell lines. However, in certain tumours the content of these subunits was high and, in view of their distinct substrate specificities and the finding that they are also over-expressed in drug resistant cell lines, should also be seriously considered in relation to intrinsic and acquired drug resistance.

J.D.H. would like to thank the MRC for financial support (grant no. G8520239).

### References

ADAMS, D.J., CARMICHAEL, J. & WOLF, C.R. (1985). Altered mouse bone marrow glutathione and glutathione transferase levels in response to cytotoxins. *Cancer Res.*, **45**, 1669.

AWASTHI, Y.C., SINGH, S.V., AHMAD, H. MOLLER, P.C. & GUPTA, V. (1988). Expression of glutathione S-transferase isozymes in human small cell lung cancer cell lines. *Carcinogenesis*, **9**, 89.

BATIST, G., TULPULE, A., SINHA, B.K., KATKI, A.G., MYERS, C.E. & COWAN, H. (1986). Overexpression of a novel anionic glutathione S-transferase in multidrug resistant human breast cells. *J. Biol. Chem.*, **261**, 15544.

BENSON, A.M., BATZINGER, R.F., OU, S.L., BUREDING, E., CHA, Y. & TALALAY, P. (1978). Elevation of hepatic glutathione S-transferase activities and protection against mutagenic metabolites of benzo(a)anthracene by dietary antioxidants. *Cancer Res.*, **38**, 4486.

BENSON, A.M. & BARRETTO, P.B. (1985). Effects of disulfiram, diethyldithiocarbamate, bisethylxanthogen and benzyliosothiocyanate on glutathione-S-transferase activities in mouse organs. *Cancer Res.*, **45**, 4219.

BUCHMANNN, A., KUHLMAN, W., SCHWARTZ, M. and 5 others (1985). Regulation and expression of four cytochrome P450 isoenzymes, NADPH cytochrome P450 reductase, glutathione S-transferase B and C and microsomal epoxide hydrolase in preneoplastic and neoplastic lesions in the rat liver. *Carcinogenesis*, **6**, 513.

BULLER, A.L., CLAPPER, M.L. & TEW, K.D. (1987). Glutathione S-transferases in nitrogen mustard-resistant and sensitive cell lines. *Molec. Pharmacol.*, **31**, 575.

CARMICHAEL, J., ADAMS, D.J., ANSELL, J. & WOLF, C.R. (1986). Glutathione and glutathione transferase levels in mouse granulocytes following cyclophosphamide administration. *Cancer Res.*, **46**, 735.

CARMICHAEL. J., DEGRAFF, W.G., GAZDAR, A.F., MINNA, J.D. & MITCHELL, J.B. (1987). Evaluation of a tetrazolium-based semi-automated colorimetric assay – assessment of chemosensitivity testing. *Cancer Res.*, **47**, 936.

CARMICHAEL, J., FORRESTER, L.M., LEWIS, A.D., HAYES, J.D. & WOLF, C.R. (1988). Glutathione S-transferase isoenzymes and glutathione peroxidase activity in normal and tumour samples from human lung. *Carcinogenesis*, **9**, 1617.

CHASSEAUD, L.F. (1979). The role of glutathione and glutathione-S-transferase in the metabolism of chemical carcinogens and other electrophilic agents. *Adv. Cancer Res.*, **29**, 175.

DI ILIO, C., SACCHETTA, P., DEL BOCCIO, G., LA ROVERE, G. & FREDERICHI, G. (1985). Glutathione peroxidase, glutathione S-transferase and glutathione reductase activity in normal and neoplastic breast tissue. *Cancer Lett.*, **29**, 39.

DI ILIO, C., DEL BOCCIO, G., ACETO, A., CASACCIA, R., MUCILLIA, F. & FEDERICI, G. (1988). Elevation of glutathione transferase activity in human lung tumor. *Carcinogenesis*, **9**, 335.

DULIK, D.M., FENESELAU, C. & HILTON, J. (1986). Characterisation of Melphalan-glutathione adducts whose formation is catalysed by glutathione S-transferases. *Biochem. Pharmacol.*, **35**, 3405.

EVANS, C.G., BODELL, W., TOKUDA, K., DOUNNE-SETZER, P. & SMITH, M. (1987). Glutathione and related enzymes in rat brain tumour cell resistance to 1,3-*bis*(2-chloroethyl)-1-nitrosourea and nitrogen mustard. *Cancer Res.*, **47**, 2525.

HABIG, W.H., BABIST, M.J. & JACKOBY, W.B. (1974). Glutathione S-transferases the first enzymatic step in mercapturic acid formation. *J. Biol. Chem.*, **249**, 7130.

HAYES, J.D., GILLIGAN, D., CHAPMAN, B.J. & BECKETT, G.J. (1983). Purification of human hepatic glutathione S-transferase and their development of a radioimmunoassay for their measurement in plasma. *Clin. Chim. Acta*, **134**, 107.

HAYES, J.D. & WOLF, C.R. (1988). Role of glutathione transferases in drug resistance. In *Glutathione Conjugation: Mechanisms and Biological Significance*, Sies, H. & Ketterer, B. (eds) p. 315. Academic Press: New York.

KITAHARA, A., SATOH, K., NISHIMURA, K. and 5 others (1984). Changes in molecular forms of rat hepatic glutathione S-transferase during chemical hepatocarcinogenesis. *Cancer Res.*, **44**, 2698.

KODATE, C., FUKUSHI, A., NARITA, T., KUDO, H., SOMA, Y. & SATO, K. (1986). Human placental form of glutathione-S-transferase (GST $\pi$) as a new immunohistochemical marker for human colonic carcinoma. *Jpn. J. Cancer Res. (Gann)*, **77**, 226.

LEWIS, A.D., HAYES, J.D. & WOLF, C.R. (1988*a*). Glutathione and glutathione-dependent enzymes in ovarian adenocarcinoma cell lines derived from a patient before and after the onset of drug resistance: intrinsic differences and cell cycle effects. *Carcinogenesis*, **9**, 1283.

LEWIS, A.D., HICKSON, I.D., ROBSON, C.N. and 7 others (1988*b*). Amplification and increased expression of alpha class glutathione S-transferase genes associated with resistance to nitrogen mustards. *Proc. Natl Acad. Sci. USA*, **85**, 8511.

LOWRY, O.H., ROSENBROUGH, N.J., FARR, A.L. & RANDALL, R.J. (1975). Protein measurements with folin phenol reagent. *J. Biol. Chem.*, **193**, 265.

McGOWAN, A.T. & FOX, B.W. (1986). A proposed mechanism of resistance to cyclophosphamide mustard in a Yoshida cell line *in vitro*. *Cancer Chemother. Pharmacol.*, **17**, 223.

MANNERVIK, B. (1985). The isoenzymes of glutathione transferase. *Adv. Enzymol.*, **57**, 357.

PICKETT, C.B., TELAKOWSKI-HOPKINS, L.A., DING, G.J.-F., ARGENBRECHT, L. & LU, A.Y.H. (1984). Complete nucleotide sequence of a glutathione S-transferase mRNA and the regulation of Ya, Yb and Yc mRNA's by 3-methylcholanthrene and phenobarbital. *J. Biol. Chem.*, **259**, 5182.

ROBSON, C.N., LEWIS, A.D., WOLF, C.R and 4 others (1987). Reduced levels of drug induced DNA cross-linking in nitrogen mustard resistant Chinese hamster ovary cells expressing elevated glutathione S-transferase activity. *Cancer Res.*, **47**, 6022.

SHEA, T.C., KELLY, S.L. & HENNER, W.D. (1988). Identification of an anionic form of glutathione transferase present in many human tumours and human tumour cell lines. *Cancer Res.*, **48**, 527.

STOCKMAN, P.K., BECKETT, G.J. & HAYES, J.D. (1985). Identification of a basic hybrid glutathione S-transferase from human liver. *Biochem. J.*, **227**, 457.

STOCKMAN, P.K., McLELLAN, L.I. & HAYES, J.D. (1987). Characterisation of the basic glutathione S-transferase B1 and B2 subunits from human liver. *Biochem. J.*, **244**, 55.

TEW, K.D., BOMBER, A.M. & HOFFMAN, S.J. (1988). *Cancer Res.*, **48**, 3622.

TOWBIN, H., STAEHELIN, T. & GORDON, J. (1979). Electrophoretic transfer of protein from polyacrylamide gels to nitrocellulose sheets: procedures and some applications. *Proc. Natl Acad. Sci. USA*, **76**, 4350.

WANG, A.L. & TEW, K.D. (1985). Increased glutathione S-transferase activity in a cell line with acquired resistance to nitrogen mustards. *Cancer Treat. Rep.*, **69**, 677.

WOLF, C.R., HAYWARD, I.P., LAWRIE, S.S. and 6 others (1987*a*). Cellular heterogeneity and drug resistance in two ovarian adenocarcinoma cell lines derived from a single patient. *Int. J. Cancer*, **39**, 695.

WOLF, C.R., LEWIS, A.D., CARMICHAEL, J. and 7 others (1987*b*). Glutathione S-transferase expression in normal and tumour cells resistant to cytotoxic drugs. In *Glutathione S-Transferases and Carcinogenesis*, Mantle, T.J., Pickett, C.B. & Hayes, J.D. (eds) p. 199. Taylor and Francis: London.